# Loss of Frmd5 Inhibits Jak2-Stat3 Signalling Pathway and Impairs Cell Apoptosis During Vagina Luminal Formation in Puberty Mice

**DOI:** 10.7150/ijbs.126669

**Published:** 2026-03-25

**Authors:** Tiantian Su, Zhenbin Wang, Yunjiao Wu, Qianchen Li, Li Tian, Xiaowei Li, Jing Zhang, Miao Yu, Yuqi Xing, Juan Du, Lijun Zhao, Jun Zhan, Hongquan Zhang

**Affiliations:** 1 Program for Cancer and Cell Biology, Department of Human Anatomy, Histology and Embryology, School of Basic Medical Sciences; Peking University International Cancer Institute; Peking University Health Science Center, 100191 Beijing, China.; 2 Department of Obstetrics and Gynecology, Reproductive Medical Center, Peking University People's Hospital, Beijing 100044, China.; 3 Department of Obstetrics and Gynecology, Peking University People's Hospital, Beijing 100044, China.; 4 Department of Obstetrics and Gynecology, Peking University First Hospital, Beijing 100034, China.

**Keywords:** Frmd5, Jak2-Stat3 signaling, apoptosis, vaginal development, longitudinal vaginal septum

## Abstract

FRMD5, FERM-domain protein 5, has been reported to be associated with tumors progession and neurodevelopment, however its molecular mechanisms in normal cells and its functions during urinary epithelium development remain unknown. In this study, we identified that Frmd5 interacts with Jak2 and Stat3, leading to the enhanced Jak2/Stat3 complex formation and subsquently promoting phosphorylation of Stat3. In a urinary epithelium specific knockout of *Frmd5* mouse, Jak2-Stat3 signaling pathway was significantly inactivated and apoptosis of epithelium was significantly downregulated compared with *Cdh16-Cre-*; *Frmd5^flox/flox^*control mouse. In *Cdh16-Cre+*; *Frmd5^flox/flox^* vaginal epithelium pro-apoptotic genes (*Casp3*, *Casp8*) was decreased and anti-apoptotic genes (*Bcl2*, *Bcl-XL*) was increased compared with *Cdh16-Cre-*; *Frmd5^flox/flox^* vaginal epithelium. A total of 56.7% *Cdh16-Cre+*; *Frmd5^flox/flox^* mice failed to form vaginal lumen and developed longitudinal vaginal septum coupled with infertility in these mice. In summary, we demonstrated that Frmd5 is essential for activation of Jak2-Stat3 signaling pathway and is required for vaginal lumen development in mice.

## Introduction

FERM domain-containing proteins are critical scaffold proteins that regulate cellular processes such as apoptosis, proliferation, and differentiation, primarily through interactions with other proteins 1, 2. To date, more than 50 FERM domain-containing proteins have been identified 3-5. Among them, FERM domain-containing protein 5 (FRMD5) is still poorly characterized, and its physiological functions have not been extensively explored. Recent studies on FRMD5 have focused mainly on its role in tumor progression 6, 7. For example, FRMD5 suppresses tumorigenesis in lung cancer H1299 cells by stabilising E-cadherin through interaction with p120-catenin 8, and it inhibits the epithelial-mesenchymal transition (EMT) and cellular motility via interaction with ROCK1 9. In papillary thyroid carcinoma, FRMD5 overexpression is associated with tumour invasiveness and metastatic potential, indicating its involvement in malignant progression 10. Our previous study demonstrated that Frmd5 deficiency leads to neurodevelopmental dysfunction and autism spectrum disorder (ASD)-like behaviour in mice 11. This phenotype is consistent with clinical observations in patients with FRMD5 mutations, who exhibit delayed motor development and intellectual disability 12. Moreover, we observed reduced fertility in *Frmd5*-deficient mice, suggesting a potential role of FRMD5 in reproductive biology. To explore the molecular function of Frmd5 in the reproductive system, we performed a proteomic screen and found that its interacting proteins were significantly enriched in the Jak2-Stat3 signaling pathway.

The JAK-STAT signaling pathway mediates a variety of biological processes, including cell apoptosis, cell proliferation, differentiation, and migration 13, 14. Within this pathway, activated JAK2 phosphorylates STAT3^15^, which then dimerizes and translocates to the nucleus. There, it regulates the expression of genes such as *Caspase-3 (Casp3), Caspase-8 (Casp8), Bcl2, Bax,* and *Bak,* thereby influencing the critical balance between cell survival and death 16-18. The precise regulation of Jak2-Stat3 signaling is crucial for organ development and tissue remodeling. For instance, studies in *Drosophila* have revealed that the Jak-Stat pathway acts as a central regulator of morphogenesis, controlling both organ size and cell fate specification by modulating the production and signaling of critical morphogens 19. During murine palatal fusion, the Jak-Stat pathway orchestrates the timely removal of the medial edge epithelium, a critical step in preventing cleft palate^20^.

Infertility is a global health issue that profoundly impacts quality of life and psychological well-being 21, 22. Among its diverse etiologies, developmental abnormalities of the reproductive system resulting from genetic defects are a significant factor. Vaginal developmental abnormalities, such as Mayer-Rokitansky-Küster-Hauser (MRKH) syndrome, represent a major cause of infertility 23, 24, with congenital vaginal agenesis occurring in approximately 1 in 4,000-5,000 female births 25. Normal vaginal morphogenesis originates from the Müllerian ducts 26 and depends on a massive wave of apoptosis during postnatal tissue remodeling to form a lumen, a process termed vaginal opening 27. Apoptosis of intermediate epithelial cells is the driving force behind this lumen formation 28. If this apoptotic process is blocked and the central epithelial cells are not cleared in a timely manner, vaginal canalization fails, resulting in congenital malformations such as transverse or longitudinal vaginal septa. The critical role of apoptosis is highlighted by studies showing that overexpression of the anti-apoptotic gene *Bcl2*^29^ ^30^ or the double knockout of pro-apoptotic genes *Bax* and *Bak* 31 both lead to a closed vaginal defect in mice. Despite this knowledge, the specific molecular mechanisms that orchestrate this apoptotic program during vaginal morphogenesis remain elusive.

In this study, we demonstrate that Frmd5 supports normal vaginal formation by modulating Jak2-Stat3 signaling. Frmd5 acts as a novel interacting protein of Jak2 and Stat3, functioning as a molecular scaffold that enhances their interaction and promotes Stat3 phosphorylation and activation. We further show that Frmd5-mediated activation of the Jak2-Stat3 pathway is essential for epithelial apoptosis during postnatal tissue remodeling. Loss of Frmd5 disrupts this apoptotic process, resulting in the formation of a longitudinal vaginal septum coupled with infertility. Our findings not only uncover a novel function for Frmd5 in Jak2-Stat3 signal transduction but also provide new insights into the molecular pathogenesis underlying congenital malformations of the reproductive tract, with potential clinical relevance.

## Materials and Methods

### Mice and genotyping

All animal experiments were approved by the Peking University Animal Care and Use Committee. We bought *Cdh16-Cre+* mice, *Frmd5^flox/flox^* and *Frmd5*-e(3xFlag-GGS) transgenic mice from Shanghai Model Organisms Center, Inc. (Shanghai, China) Genotyping was determined via PCR using the following primers: *Frmd5* knockout mice (*Frmd5*^-/-^): M13F-FAM 5'-TGTAAAACGACGGCCAGT-3', T3-SENSE 5'-TGTAAAACGACGGCCAGTTACTTGTGGCCTCCGAGAAC-3', T3-ANTISENSE 5'-GGATCACCATTGCTGGGAGT-3'; *Frmd5^flox/flox^*: forward 5'-ATTCTGAAATCTCATCAGTACTTT-3', reverse 5'-CCTAGCTTCAGGTGCCACTCTCAG-3'; *Cdh16-Cre+*: forward 5'-ATTTGCCTGCATTACCGGTCG-3', reverse 5'-CAGCATTGCTGTCACTTGGTC-3'; *Frmd5*-e(3xFlag-GGS): forward 5'-CTTGCTTGGGGTTTGGGG-3', reverse 5'-TGCAGCTGTACTCGCGCTC-3'.

### Cell culture

HeLa cell lines and human embryonic kidney HEK293T cell lines were cultured in Dulbecco's Modified Eagle Medium (DMEM) containing 10% foetal bovine serum (Invitrogen, Carlsbad, CA, USA), supplemented with 100 mg/mL streptomycin and 100 units/mL penicillin. These cell lines were purchased from the American Type Culture Collection (Manassas, VA, USA) and maintained at 37°C in a 5% CO₂ incubator.

### RNA extraction and RT-qPCR

Total RNA was extracted using Trizol reagent (Invitrogen) according to the manufacturer's protocol, and 1 mg of RNA was reverse transcribed to cDNA using HiScript II Q RT SuperMix for qPCR (+gDNA cursor) (Vazyme, Nanjing, China). Relative mRNA levels were detected using RT-qPCR, performed in triplicate, and normalised to glyceraldehyde 3-phosphate dehydrogenase (*Gapdh*) expression levels. The reverse-transcription products were amplified using LightCycler 96 (Roche, Basel, Switzerland) and the data were analysed using GraphPad Prism v9.0 (GraphPad Software, La Jolla, CA, USA). All primers used in this analysis are listed in Table S1.

### RNA-seq analysis

Vaginal tissues were collected from *Cdh16-Cre-*; *Frmd5^flox/flox^* and *Cdh16-Cre+*; *Frmd5^flox/flox^* mice. The tissues were thoroughly homogenised on ice, and RNA was extracted using Trizol reagent (Invitrogen) following the manufacturer's protocol. Sequencing was performed on the Hiseq-PE150 platform (Novogene, Beijing, China). Quantification was performed using FeatureCounts v1.5.0-P3 and fragments per kilobase of transcript per million mapped reads (FPKM) values were obtained. The resulting matrix contained raw gene counts for each gene and each sample.

### Western blot analysis

Cell lysates or tissues were treated with radioimmunoprecipitation assay (RIPA) lysis buffer supplemented with 1× Roche PhosSTOP phosphatase inhibitor cocktail (Roche) and 1× inhibitor cocktail (Roche). Aliquots (20 μg) of protein were separated by sodium dodecyl sulphate polyacrylamide gel electrophoresis (SDS-PAGE) and transferred to the polyvinylidene fluoride membrane. Membranes were blocked in 5% non-fat powdered milk for 1 h at room temperature and incubated with specific antibodies overnight at 4°C. The antibodies used for Western blot analysis in this study included rabbit anti-FRMD5 (YN3224, 1:1000 dilution; Immunoway, San Jose, CA, USA); mice anti-GAPDH (TA-08, 1:1000 dilution; Zhong Shan Jin Qiao, Guangzhou, China); rabbit anti-DDDDK tag (ab205606, 1:1000 dilution; Abcam, Cambridge, UK); rabbit anti-Jak2 (ab108596, 1:1000 dilution; Abcam); rabbit anti-Stat3 (ab68153, 1:1000 dilution; Abcam); rabbit anti-pStat3-Y705 (ab76315, 1:1000 dilution; Abcam); and rabbit anti-pStat3-S727 (ab32143, 1:1000 dilution; Abcam). The membranes were washed three times in tris-buffered saline with Tween20 (TBST) buffer, and incubated with appropriate horseradish peroxidase (HRP)-conjugated secondary antibodies for 1 h at room temperature. Immobilised antibodies were detected using a Super Signal Chemiluminescence kit (Thermo Fisher Scientific, Waltham, MA, USA).

### Mass spectrometry analysis

Vaginal tissues from mice were thoroughly homogenised on ice. The tissues were lysed on ice for 30 min using RIPA buffer (1× phosphate-buffered saline [PBS], pH 7.4, 0.5% sodium deoxycholate, 1% Triton X-100, and 0.1% SDS) with protease inhibitor cocktail (Roche). The samples were then centrifuged at 12,000 rpm for 15 min at 4°C, and the supernatant was collected. Protein concentrations were measured using a BCA Protein Assay Kit (Thermo Fisher Scientific) to ensure comparable protein levels across groups. Co-IP assays were performed by rotating the samples with Flag M2 beads (Sigma-Aldrich, St. Louis, MO, USA) at 4 °C for 6 h. After extensive washing, the protein samples were eluted by boiling in SDS sample buffer at 99 °C for 10 min. The samples were separated by SDS-PAGE, followed by staining with Coomassie Brilliant Blue for visualisation; then, mass spectrometry analysis was performed.

Gel slices corresponding to the protein bands of interest were excised and subjected to in-gel digestion with sequencing-grade trypsin. The resulting peptides were extracted and analyzed by liquid chromatography-tandem mass spectrometry (LC-MS/MS). Peptide separation was performed on a Thermo Scientific Ultimate 3000 nano-UPLC system coupled online to an Orbitrap Fusion Lumos mass spectrometer (Thermo Fisher Scientific). Peptides were loaded onto a reversed-phase analytical column (Acclaim PepMap RSLC, 75 μm × 250 mm, C18) and eluted using a 120-min linear gradient at a flow rate of 0.30 μL/min, with mobile phase A consisting of 0.1% formic acid in water and mobile phase B consisting of acetonitrile containing 0.1% formic acid. The mass spectrometer was operated in data-dependent acquisition mode using Xcalibur software (version 4.1.50). Full MS scans were acquired in the Orbitrap over a mass range of 375-1500 m/z at a resolution of 60,000, followed by MS/MS scans of selected precursor ions. Raw data were processed and searched against the UniProt mouse protein database using Proteome Discoverer software (version 2.4, Thermo Fisher Scientific) for protein identification.

### Immunoprecipitation

HeLa cell lysates and *Frmd5* knockout mice (*Frmd5*^-/-^) tissues were treated with RIPA lysis buffer supplemented with 1× Roche PhosSTOP phosphatase inhibitor cocktail (Roche) and 1× inhibitor cocktail (Roche). The cells were subjected to centrifugation at 12,000 rpm for 15 min at 4°C, and the supernatant was collected. After adjusting the protein concentration consistently, the precleared lysates were incubated with the indicated antibody overnight at 4°C. The lysates were incubated with 40 μL of Protein A agarose (Santa Cruz Biotechnology, Dallas, TX, USA) for 8 h at 4°C with rotation. The complex was washed six times with RIPA lysis buffer, resuspended with 60 μL of 2× loading buffer, and boiled at 100°C for 8 min prior to Western blot analysis.

### GST-pull down assay

Transfected HEK293T cells were lysed on ice for more than 30 min. Purified GST or GST fusion proteins were immobilised on Glutathione Sepharose 4B beads (Sigma-Aldrich). The beads were incubated with pre-cleared HEK293T cell extracts overnight at 4°C, and then washed six times with ice-cold RIPA, resuspended with 80 μL of 2× loading buffer, and boiled at 100°C for 8 min prior to Western blot analysis.

### Histology and immunohistochemistry

Mouse vaginal tissue was fixed with 4% buffered formalin (Thermo Fisher Scientific), embedded in paraffin, and sectioned to 5 μm prior to immunohistochemical or H&E staining for protein detection or structural examination, respectively. Briefly, the sections were deparaffinised with xylene and rehydrated with ethanol. Sections were blocked using 3% hydrogen peroxide for 15 min at room temperature. After washing three times in PBS (pH 7.6), the slides were heated and antigen retrieval was performed in 0.01 M citrate buffer (pH 6.0) in a 100 °C water bath for 30 min. Then, the slides were cooled to room temperature and washed three times in PBS (pH 7.6) and non-specific binding of antibodies was blocked using 10% normal goat serum for 20 min. The sections were probed overnight with anti-*Frmd5* (YN3224, 1:100 dilution; Immunoway), anti-DDDDK tag (ab205606, 1:200 dilution; Abcam), anti-Jak2 (3230T, 1:200 dilution; Cell Signalling Technology), anti-Stat3 (ab68153, 1:200 dilution; Abcam), anti-pStat3-Y705 (ab76315, 1:100 dilution; Abcam), anti-pStat3-S727 (ab32143, 1:250 dilution; Abcam), or anti-CK14 (10143-1-AP, 1:200 dilution; Proteintech, Rosemont, IL, USA). The sections were washed three times in PBS (pH 7.6), incubated with biotinylated IgG (PV6001; Zhong Shan Jin Qiao) for 45 min at room temperature, and then washed three times in PBS (pH 7.6). Using diaminobenzidine as a substrate and haematoxylin as a counterstain, the sections were examined and photographed using a microscope (BX51; Olympus, Tokyo, Japan).

### Quantitative analysis of tissue section images

Multi-colour staining was performed using commercially available kits (TGFP550; TissueGnostics, Vienna, Austria and AXT37025031; AlphaTSA; Alpha X Biotech, Beijing, China) according to the manufacturers' instructions. Total cells were identified according to 4′,6-diamidino-2-phenylindole (DAPI) staining. Immunofluorescence images were scanned using a microscope slide scanner (Axio scan 7; Zeiss, Oberkochen, Germany). Immunofluorescence image analysis was performed using the Visiopharm Image Analytical System v5.0.3.1309. Tissue edges and tissue-free areas were identified using VisioPharm software to define a region of interest corresponding to the vaginal epithelium. For quantitative analysis, the mean fluorescence intensity of each target marker was calculated on a per-cell basis within the defined epithelial region of interest and compared between experimental groups.

### TUNEL staining

Vaginal tissues from mice were collected and paraffin-embedded sections were prepared as previously described. Apoptotic cells were detected via a TUNEL assay (Roche Applied Science), following the manufacturer's instructions. Sections were counterstained with DAPI to identify cell nuclei. Both TUNEL-positive (green) and TUNEL-negative (blue) nuclei were counted.

### Statistical analysis

Statistical analysis was performed using GraphPad Prism v9.0 software (GraphPad Software). All results are expressed as means ± standard error of the mean or standard deviation. Quantitative RT-PCR analysis was performed using unpaired one- or two-tailed Student's *t*-tests. Statistical significance was evaluated at a threshold of *P* < 0.05. All data were tested for homogeneity of variance prior to selecting a parametric or nonparametric test for analysis.

## Results

### Frmd5-interacting proteins are enriched in the Jak2-Stat3 signaling pathway *in vivo*

**Figure 1:**
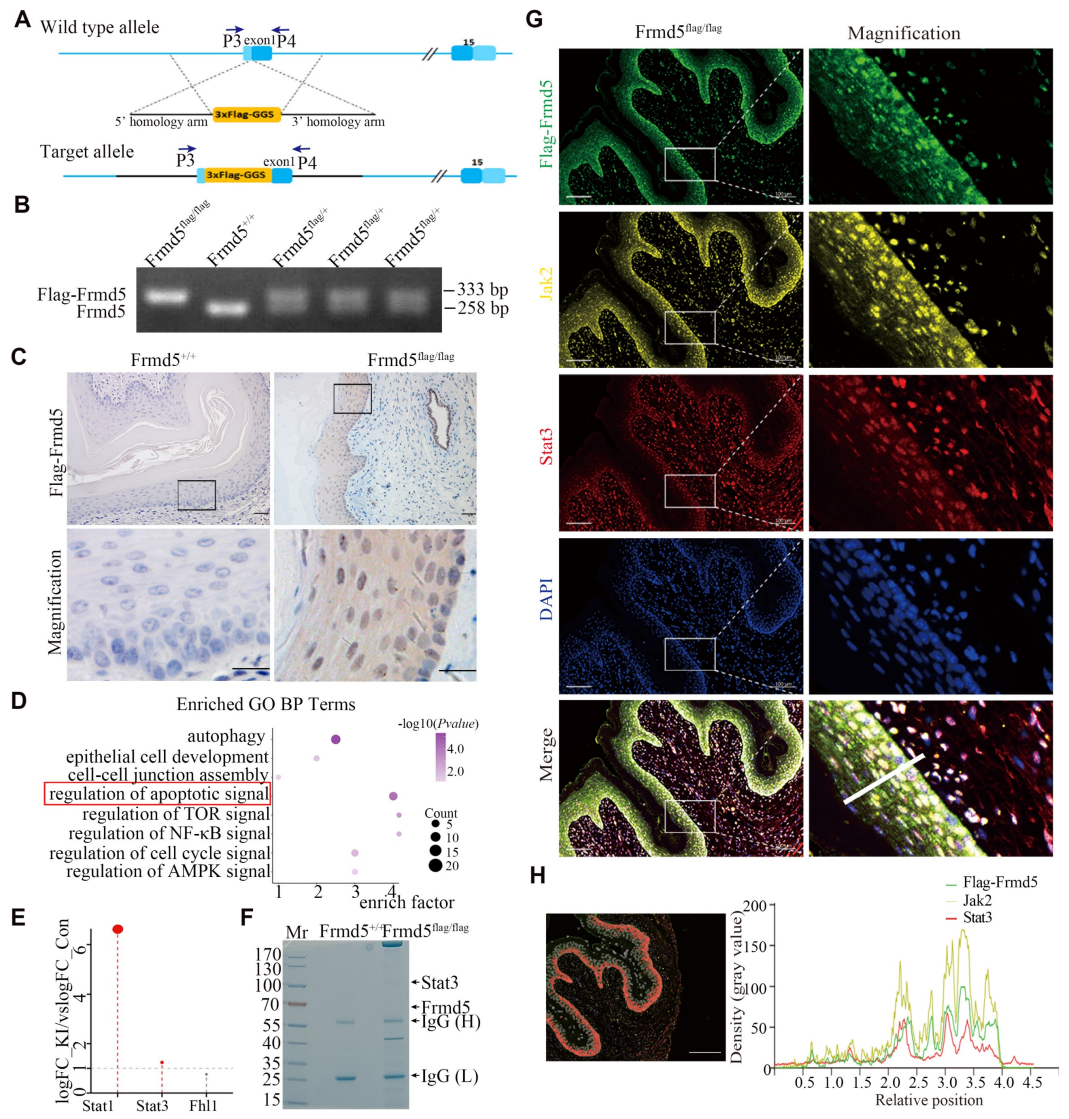
Frmd5-interacting proteins are enriched in the Jak2-Stat3 signaling pathway *in vivo***.** (A) Schematic diagram of the *Frmd5* gene structure in the Flag-tagged mice model. Blue arrows indicate the positions of primers used for genotyping. (B) PCR genotyping results showing elongated amplification products following insertion of the Flag sequence into the *Frmd5* locus. (C) IHC analysis of the distribution of the Flag-Frmd5 protein. (D) Mass spectrometry and Gene Ontology enrichment analysis of Frmd5-interacting proteins isolated from vaginal tissues of *Frmd5^+/+^* and *Frmd5^flag/flag^* mice. (E) Analysis of apoptosis-related proteins identified in in the proteomic dataset, highlighting components of Jak-Stat signaling pathway. (F) Co-IP of vaginal tissue proteins from *Frmd5^+/+^* and *Frmd5^flag/flag^* mice, visualized by Coomassie Blue staining, confirming the presence of Flag-Frmd5 and Stat3. (G) Multiplex immunofluorescence analysis showing the distribution of Jak2, Stat3, and Frmd5 in the vaginal epithelium. Scale bar: 100 μm. (H) Red markers highlight epithelial cells exhibiting strong signals of all three proteins. The fluorescence intensity profile along the white line in panel G is shown. The x-axis represents cell position, and the y-axis indicates fluorescence intensity. Scale bar: 200 μm.

To investigate the molecular mechanism of Frmd5, we generated *Frmd5* knock-in mice carrying a Flag tag. The Flag tag was inserted upstream of the first exon of *Frmd5* (Fig. 1A). Genotyping was confirmed via PCR using the P3 and P4 primers. WT C57BL/6J mice (*Frmd5^+/+^*) showed a single 258-bp band, heterozygous mice (*Frmd5^flag/+^*) displayed both 258-bp and 333-bp bands, and homozygous knock-in mice (*Frmd5^flag/flag^*) showed a 333-bp band (Fig. 1B). Our previous work has shown that Frmd5 influences reproductive system function in mice. Given the scaffold role of FERM-domain proteins in coordinating proliferation and apoptosis, and the intense cellular remodeling of the vaginal epithelium, we prioritized the vagina—where endogenous Frmd5 is strongly expressed (Fig. S1)—as the tissue for interaction profiling and functional interrogation. Immunohistochemistry revealed that Flag-Frmd5 was localized in both cytoplasm and nucleus of vaginal epithelial cells in *Frmd5^flag/flag^* mice, which mirrored the distribution of endogenous Frmd5, whereas no signal was observed in *Frmd5^+/+^* mice (Fig. 1C). These results confirm the successful generation of Flag-tagged *Frmd5* knock-in mice.

Proteins extracted from vaginal tissues of *Frmd5^+/+^* and *Frmd5^flag/flag^* mice were analyzed by mass spectrometry. Gene Ontology (GO) enrichment analysis of the proteomics data revealed significant enrichment of apoptosis-related pathways (Fig. 1D). Among apoptosis-related proteins, Stat1, Stat3, and Fhl1 were mapped to the Jak-Stat pathway (Fig. 1E). While Stat1 was expressed in the epithelium, its phosphorylated form was undetectable (Fig. S2), so we focused on Stat3. Coomassie-stained gels analyzed by mass spectrometry showed significant enrichment of Stat3 at approximately 88 kDa in *Frmd5^flag/flag^* mice, consistent with its predicted molecular weight (Fig. 1F). To examine their spatial localization* in vivo*, multicolor immunofluorescence revealed that Flag-Frmd5, Jak2, and Stat3 are present within vaginal epithelial cells from *Frmd5^flag/flag^*mice (Fig. 1G, H). These findings indicate that Flag-Frmd5, Jak2, and Stat3 are expressed in the same epithelial cells population and display overlapping distribution patterns. These data further support the involvement of Frmd5 proteins in the Jak2-Stat3 signaling pathway *in vivo*, suggesting that Frmd5 may regulate epithelial function through this pathway.

### Frmd5 directly binds Jak2 and Stat3 via its N-terminal domain

**Figure 2:**
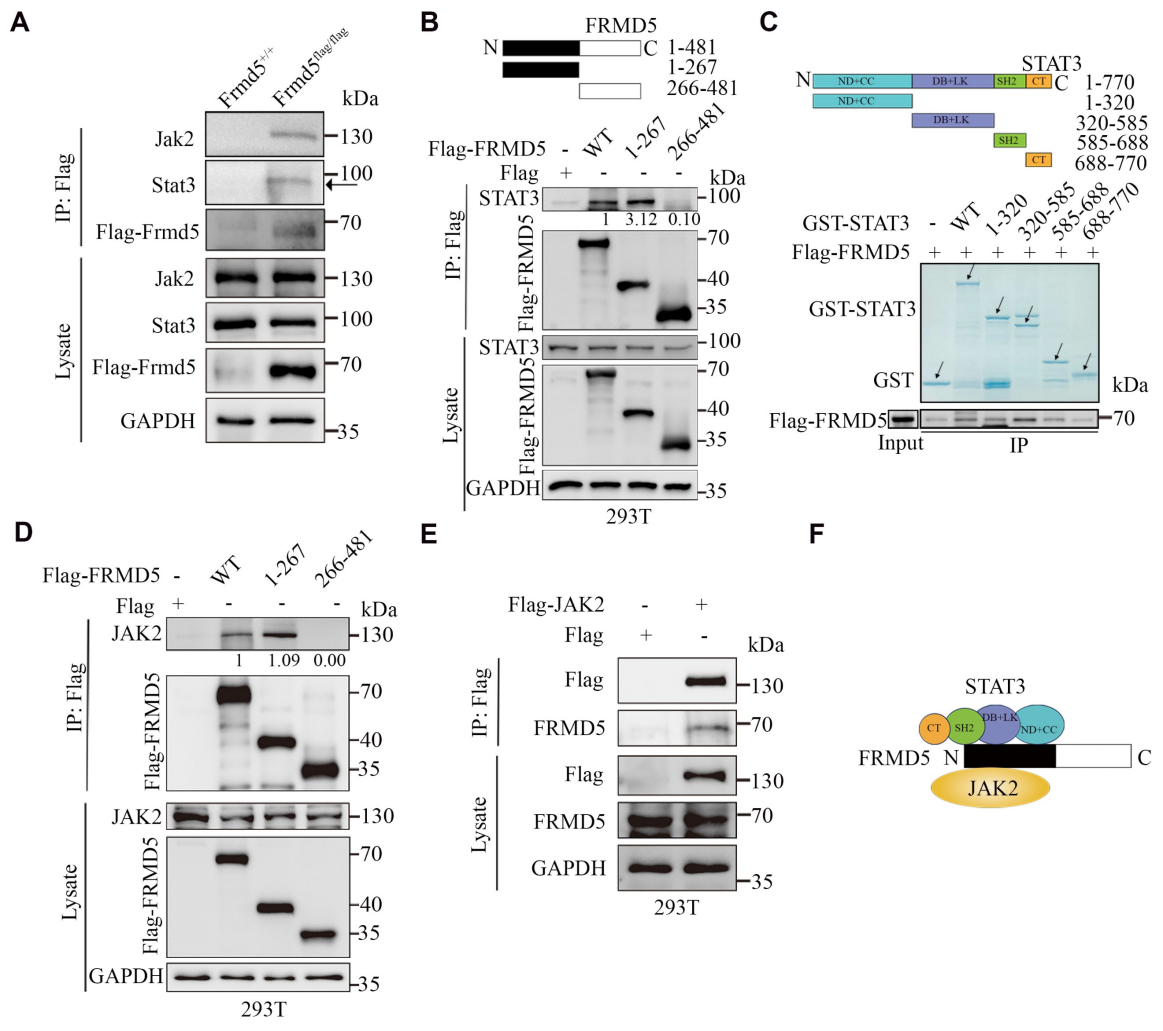
Frmd5 directly binds Jak2 and Stat3 via its N-terminal domain. (A) Co-IP experiments confirmed that Frmd5 interacts with both Jak2 and Stat3 in mice vagina. (B) Schematic diagram of FRMD5 protein segments. Co-IP analysis reveals STAT3 interacts specifically with the N-terminal region (1-267) of Frmd5. (C) Schematic diagram of STAT3 protein segments. GST pull-down assays demonstrate FRMD5 interacts with STAT3 regions 1-320, 320-585, and 586-688. Arrows indicate proteins detected at their expected molecular weights. Domain abbreviations: ND, N-terminal domain; CC, coiled-coil domain; DB, DNA-binding domain; LK, linker domain; SH2, Src homology 2 domain. (D) Co-IP assays in HEK293T cells show that JAK2 interacts with the N-terminal region (1-267) of FRMD5. (E) Co-IP assays demonstrate that FRMD5 interacts with exogenous JAK2 in HEK293T cells. (F) Schematic representation of the interaction model between FRMD5, JAK2, and STAT3. Experiments were independently repeated at least three times.

To determine the detailed molecular mechanisms by which Frmd5 regulates the Jak2-Stat3 signaling pathway, we conducted co-immunoprecipitation (Co-IP) experiments using proteins extracted from vaginal tissues of *Frmd5^+/+^* and *Frmd5^flag/flag^* mice. The results confirmed that Frmd5 interacts with Jak2 and Stat3 in the mouse vaginal tissue (Fig. 2A). To map the specific interacting domains, different constructs to express wildtype FRMD5 and its variants including full-length (1—481), N-terminal (1—267), and C-terminal (266—481) were constructed. These constructs were individually transfected into HEK293T cells, and Co-IP analysis revealed that STAT3 specifically interacted with the N-terminal region (1-267) of FRMD5 (Fig. 2B).

To further delineate the interaction sites between FRMD5 and STAT3, we performed *in vitro* glutathione-S-transferase (GST) pull-down assays. STAT3, which comprises six functional domains, was divided into five segments: full length (1-770), ND+CC (1-320), DB+LK (320-585), SH2 (585-688), and CT (688-770). For clarity, domains are abbreviated as ND (N-terminal domain), CC (coiled-coil domain), DB (DNA-binding domain), LK (linker domain), SH2 (Src homology 2 domain), and CT (C-terminal transactivation domain). GST-tagged STAT3 recombinant proteins were overexpressed, purified, and incubated with protein extracts from HEK293T cells transfected with Flag-tagged FRMD5. The results showed that FRMD5 interacted with three STAT3 segments: 1-320 (ND+CC), 320-585 (DB+LK), and 585-688 (SH2) (Fig. 2C). Subsequently, Co-IP assays demonstrated that JAK2 interacted with the N-terminal region (1-267) of FRMD5 in HEK293T cells (Fig. 2D). Additionally, FRMD5 was shown to interact with exogenous JAK2 in HEK293T cells (Fig. 2E). Together, these data indicate that FRMD5 engages both JAK2 and STAT3 predominantly via its N-terminal domain (Fig. 2F).

### Frmd5 activates the Jak2-Stat3 signaling pathway by enhancing the interaction between Jak2 and Stat3

**Figure 3:**
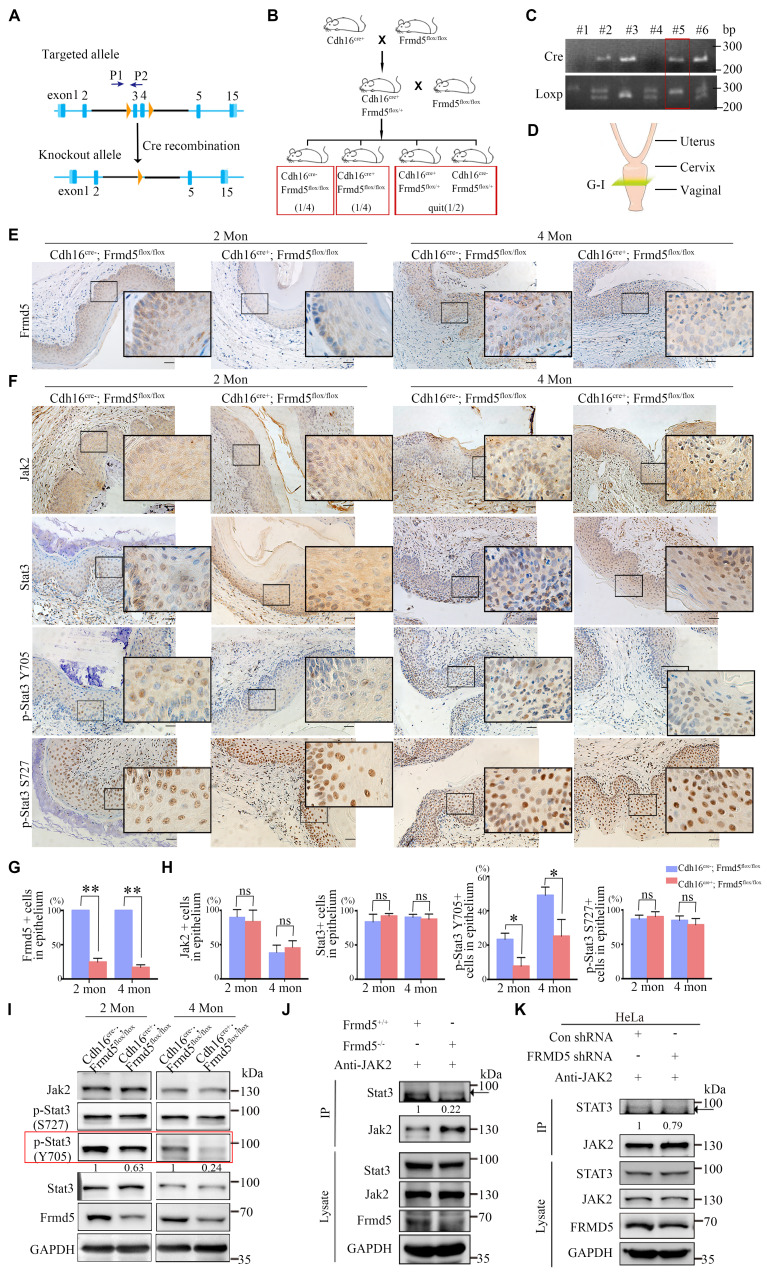
Frmd5 activates the Jak2-Stat3 signaling pathway by enhancing the interaction between Jak2 and Stat3. (A) Schematic representation of the *Frmd5* gene structure in the urinary epithelium-specific *Frmd5* knockout mice model. Blue arrows P1 and P2 indicate the positions of primers used for genotyping. (B) Schematic diagram of the breeding strategy used to generate urinary epithelium-specific *Frmd5* knockout mice. (C) PCR genotyping results. The upper panel shows the presence of the Cre recombinase gene. The lower panel shows PCR products distinguishing wild-type and floxed *Frmd5* alleles. (D) Schematic representation of the sectioning positions corresponding to panel G. (E) Immunohistochemistry analysis of Frmd5 expression in the vagina of 2-month-old and 4-month-old *Cdh16-Cre-*; *Frmd5^flox/flox^* and *Cdh16-Cre*+; *Frmd5^flox/flox^* mice. Scale bar: 50 μm in the original images, and 20 μm in the magnified views in the black boxes. (F) Immunohistochemical staining showing the distribution of Jak2, Stat3, p-Stat3 (Y705), and p-Stat3 (S727) proteins in the vaginal epithelial cells of 2-month-old and 4-month-old mice before and after epithelial-specific knockout of *Frmd5*. (G) Quantification of Frmd5-positive cells in the vaginal epithelium of 2-month-old and 4-month-old *Cdh16-Cre-*; *Frmd5^flox/flox^* and *Cdh16-Cre+*; *Frmd5^flox/flox^* mice. Data are presented as mean ± SEM. ** P < 0.01. (H) Quantification of Jak2, Stat3, p-Stat3 (Y705), and p-Stat3 (S727) positive cells in vaginal epithelial cells. (I) Western blot analysis of the expression of proteins related to the Jak2-Stat3 signaling pathway in the vagina of 2-month-old and 4-month-old mice before and after epithelial-specific knockout of *Frmd5*. (J) Co-IP analysis the impact of *Frmd5* depletion on the interaction between Jak2 and Stat3 in the intestinal tissue of *Frmd5*^-/-^ mice. (K) Co-IP analysis of the effect of *FRMD5* knockdown on the interaction between JAK2 and STAT3 in HeLa cells. Scale bars: 50 μm in the original images; 20 μm in the magnified images within the black boxes. Experiments were independently repeated at least three times. Data are presented as mean ± SEM. * *P* < 0.05.

To explore the function of Frmd5 in the urinary system, we generated a urinary epithelium-specific *Frmd5* knockout mice model using *Cdh16-Cre* mice. Prior studies have reported Cdh16 expression in the embryonic Müllerian-duct epithelium and detected *Cdh16-Cre* activity in embryonic Müllerian-duct, indicating that recombination can occur during Müllerian-duct morphogenesis. Given that the adult vaginal epithelium is derived predominantly from Müllerian-duct epithelium, this strategy enables embryonic deletion of Frmd5 in the prospective vaginal epithelial lineage, thereby achieving a urinary epithelium-specific knockout. To experimentally validate Cdh16 expression in the vaginal epithelium at postnatal stages, we performed immunohistochemical analysis of vaginal tissues from both 3-week-old and 2-month-old mice. As shown in Supplementary Figure S5, Cdh16 protein was specifically detected in the vaginal epithelium, with no detectable expression in the surrounding stromal compartment. This epithelial-restricted expression pattern was consistently observed across developmental stages, providing direct experimental support for *Cdh16-Cre* activity in vaginal epithelial cells. To generate this model, we engineered *Frmd5^flox/flox^* mice with loxP sites located after the second and fourth exons of *Frmd5* (Fig. 3A). The *Cdh16-Cre+* mice were then crossed with *Frmd5^flox/flox^* mice, resulting in urinary epithelium-specific *Frmd5* knockout mice (*Cdh16-Cre+*; *Frmd5^flox/flox^*) (Fig. 3B). Mouse genotypes were identified by polymerase chain reaction (PCR) using the P1 and P2 primers. The PCR results showed a 254-bp fragment in *Frmd5^+/+^* mice, both 254-bp and 300-bp fragments in *Frmd5*^flox/+^ mice, and a 300-bp fragment in *Frmd5^flox/flox^* mice (Fig. 3C). We used *Cdh16-Cre*^-^; *Frmd5^flox/flox^* mice as control mice and *Cdh16-Cre+*; *Frmd5^flox/flox^* mice as knockout mice in subsequent experiments. We prepared vaginal histological sections as described and shown in Fig. 3D. To further analyze histological changes after *Frmd5* deletion, these sections were examined by hematoxylin-eosin staining. Based on well-defined histological criteria, the epithelial compartment was identified as the continuous stratified squamous epithelial layer clearly demarcated from the underlying stroma. The same criteria were used consistently for all subsequent quantitative and qualitative analyses. To confirm knockout efficiency, immunohistochemistry revealed significantly reduced Frmd5 protein expression in vaginal epithelium cells of *Cdh16-Cre+*; *Frmd5^flox/flox^* mice compared with controls (Fig. 3E). Quantitative analysis further demonstrated a marked reduction in the proportion of Frmd5-positive epithelial cells in knockout mice relative to controls at both 2 and 4 months postnatally (Fig. 3G). Thus, this knockout mouse model is suitable for downstream analyses.

We examined the role of Frmd5 in modulating the Jak2-Stat3 signaling pathway by analyzing vaginal tissues from both 2- and 4-month-old mice. Immunohistochemistry and Western blot analyses were performed to assess changes in Jak2-Stat3 pathway-related proteins in vaginal tissues from* Cdh16-Cre-*; *Frmd5^flox/flox^* and* Cdh16-Cre+*; *Frmd5^flox/flox^* mice. Jak2 and Stat3 expression was detected in the cytoplasm and nuclei of most epithelial cells in 2- and 4-month-old* Cdh16-Cre-*;*Frmd5^flox/flox^* and* Cdh16-Cre+*;*Frmd5^flox/flox^* mice. No significant differences in the percentage of Jak2- or Stat3-positive epithelial cells were observed but, the proportion of p-Stat3 (Y705), the active nuclear form, was significantly reduced in *Cdh16-Cre+*;*Frmd5^flox/flox^* mice at both 2 and 4 months, while p-Stat3 (S727) remained unchanged (Fig. 3F, 3H). Western blotting further confirmed the reduction of Frmd5 and p-Stat3 (Y705) in *Cdh16-Cre+*;*Frmd5^flox/flox^* mice, while Jak2, Stat3, and p-Stat3 (S727) remained unchanged at both ages (Fig. 3I).

To determine how Frmd5 influences Stat3 phosphorylation, we investigated its effects on Jak2-Stat3 interactions. We next asked whether Frmd5 promotes Jak2-Stat3 complex formation. Co-IP analysis of intestinal tissues from global *Frmd5* knockout mice (*Frmd5*^-/-^) (Fig. S3) demonstrated a marked reduction in Jak2-Stat3 interaction following *Frmd5* depletion (Fig. 3J). Similarly, *FRMD5* knockdown in human cervical cancer (HeLa) cells significantly reduced the JAK2-STAT3 interaction (Fig. 3K). Thus, FRMD5 facilitates Stat3 (Y705) phosphorylation by stabilizing the Jak2-Stat3 interaction *in vivo* and in cells.

### *Frmd5* depletion suppresses apoptosis in mice vaginal epithelium

**Figure 4:**
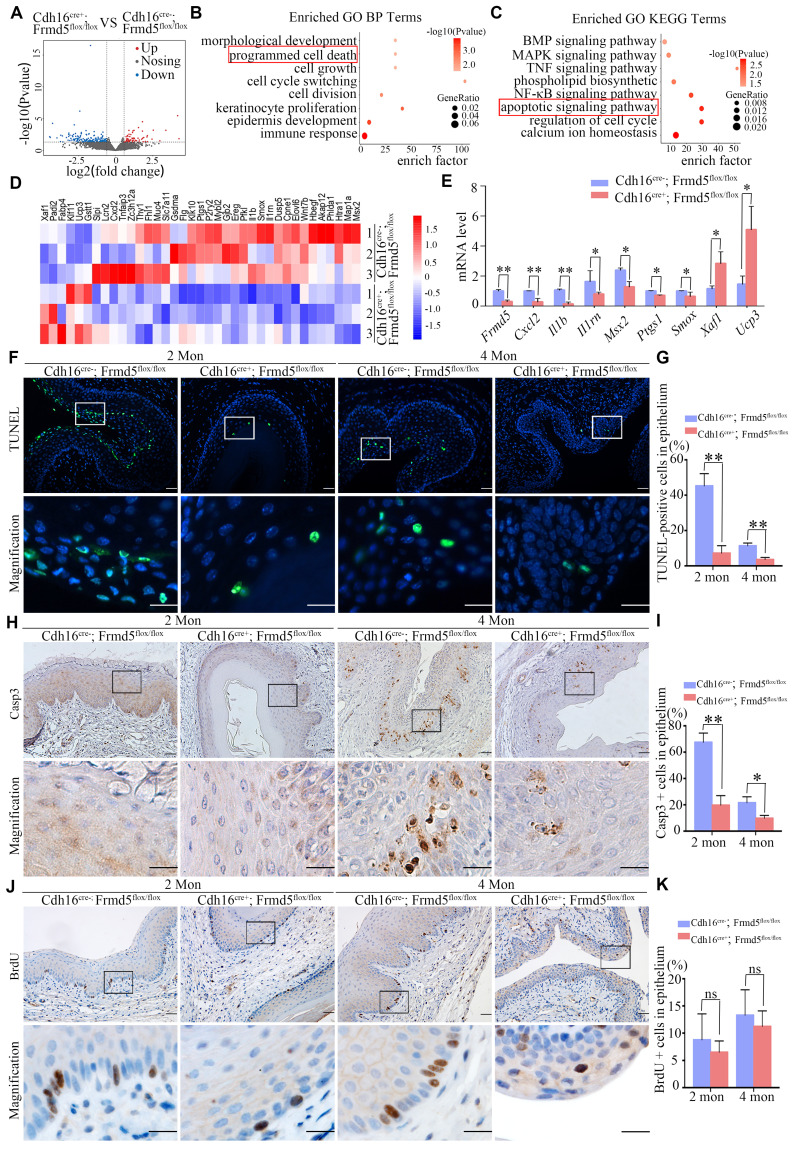
*Frmd5* depletion suppresses apoptosis in mice vaginal epithelium. (A) RNA-seq analysis of total RNA extracted from vaginal tissues of *Cdh16-Cre-*; *Frmd5^flox/flox^* and *Cdh16-Cre+*; *Frmd5^flox/flox^* mice at 2 months of age. Genes exhibiting a fold change ≥ 2 between the two groups (*Cdh16-Cre+*; *Frmd5^flox/flox^* vs. *Cdh16-Cre-*; *Frmd5^flox/flox^*) are shown in blue (downregulated) or orange (upregulated).* P* value < 0.05. (B) GO analysis of biological process enriched among genes regulated by *Frmd5*. (C) KEGG pathway enrichment analysis of genes regulated by *Frmd5*. (D) Heatmap illustrating the expression levels of apoptosis-related genes among the differentially expressed genes, with color ranging from blue to red indicating low to high gene expression levels. (E) Validation of mRNA expression changes in apoptosis-related genes using qPCR, using *Gapdh* as an internal control. (F) TUNEL staining analysis of epithelial cell apoptosis in the vagina before and after *Frmd5* knockout. (G) Quantification of TUNEL-positive cells among vaginal epithelial cells. (H) Immunostaining of Casp3 to assess epithelial cell apoptosis in the vagina before and after *Frmd5* knockout. (I) Quantification of Casp3-positive cells among vaginal epithelial cells. (J) BrdU staining analysis of epithelial cell proliferation in the vagina before and after *Frmd5* knockout. (K) Quantification of BrdU-positive cells among vaginal epithelial cells. The scale bars in F, H, and J: original images 50 μm, magnified images of the highlighted regions 20 μm. Experiments were independently repeated at least three times and statistical significance was determined by *t*-tests. Data are presented as mean ± SEM. * *P* < 0.05, ** *P* < 0.01.

We further validated the function of Frmd5 *in vivo*. To increase the robustness of our findings, we analyzed vaginal tissues from 2-month-old *Cdh16-Cre+*; *Frmd5^flox/flox^* and *Cdh16-Cre-*;*Frmd5^flox/flox^* mice by RNA sequencing (RNA-seq). A total of 95 genes were significantly downregulated and 37 were upregulated in knockout mice (Fig. 4A). GO analysis linked these differentially expressed genes to morphological development, epithelial differentiation and regulation of cell death, consistent with the fundamental biological functions of the FERM-domain protein family (Fig. 4B). Kyoto Encyclopedia of Genes and Genomes (KEGG) pathway analysis revealed disruptions in TNF, NF-κB, and MAPK signaling pathways in vaginal tissues of knockout mice (Fig. 4C), suggesting broad pathway dysregulation. We further analysed the mRNA levels of genes altered by *Frmd5* knockout using quantitative reverse-transcription PCR (RT-qPCR). As expected, *Frmd5* mRNA was markedly reduced in knockouts. Pro-apoptotic genes such as *Cxcl2, Il1b, Il1rn, Msx2, Ptgs1,* and *Smox* were downregulated, whereas anti-apoptotic genes *Xaf1* and *Ucp3* were upregulated in the knockout mice (Fig. 4D, E). These gene expression changes indicate impaired apoptotic signaling in the vaginal of *Frmd5*-deficient mice.

To validate at the cellular level, we examined survival-related markers in female mice before and after *Frmd5* knockout. We selected mice at 2 and 4 months postnatally for further analyses. Terminal deoxynucleotidyl transferase dUTP nick-end labelling (TUNEL) staining showed that TUNEL-positive cells were primarily located near the vaginal lumen (Fig. 4F). Quantification revealed a significant reduction in TUNEL-positive epithelial cells in *Cdh16-Cre+*; *Frmd5^flox/flox^* mice compared with *Cdh16-Cre-*; *Frmd5^flox/flox^* mice at both ages (Fig. 4G). An immunostaining experiment using antibody against Casp3, a marker for early apoptosis, showed predominant positive signals in epithelial cells distant from the lumen (Fig. 4H). The percentage of Casp3-positive epithelial cells was significantly lower in *Cdh16-Cre+*; *Frmd5^flox/flox^* mice than in *Cdh16-Cre-*; *Frmd5^flox/flox^* mice at both 2 and 4 months (Fig. 4I). To evaluate cell proliferation, we performed 5-bromo-2´-deoxyuridine (BrdU) labelling. BrdU-positive nuclei were present in basal epithelial cells distant from the lumen, with no significant differences among different groups (Fig. 4 J, K), suggesting that Frmd5 has no effect on cell proliferation while primarily affects apoptosis. These findings demonstrate that *Frmd5* depletion disrupts apoptosis-regulating gene expression and suppresses epithelial cell apoptosis in the vaginal epithelium.

### Depletion of *Frmd5* inhibits the activation of Stat3 and regulates the apoptosis-related genes

**Figure 5:**
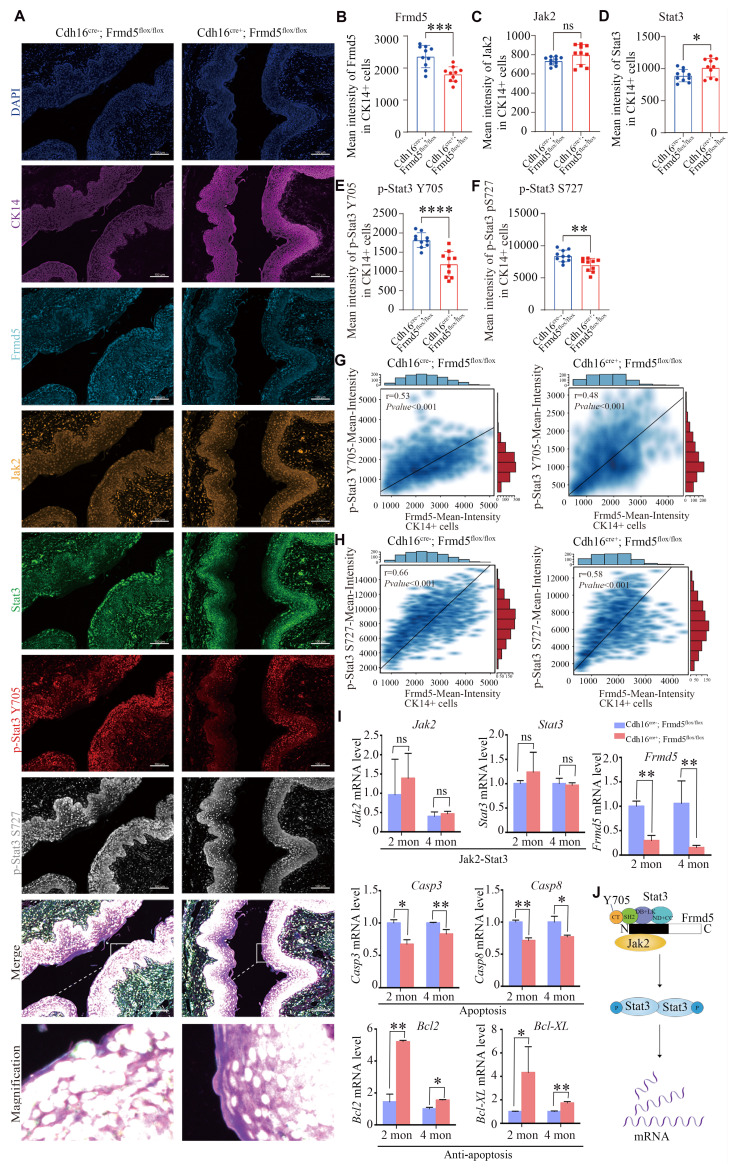
Depletion of *Frmd5* inhibits the activation of Stat3 and regulates apoptosis-related genes. (A) Multicolor immunofluorescence staining showing the localization of Frmd5, CK14, and components of the Jak2-Stat3 in vaginal tissues of 2-month-old* Cdh16-Cre-; Frmd5^flox/flox^* and* Cdh16-Cre+; Frmd5^flox/flox^* mice. (B-F) Quantitative analysis of fluorescence intensity for each target protein shown in panel A using the Visiopharm image analysis system (*n* = 10 fields of view at random per genotype). Statistical testing was performed by unpaired* t-*test. Data are presented as mean ± SD. *P < 0.05, **P < 0.01, ***P < 0.001, ****P < 0.0001. (G) Correlation analysis between Frmd5 and p-Stat3 (Y705) fluorescence intensity in individual epithelial cells. (H) Correlation analysis between Frmd5 and p-Stat3 (S727) fluorescence intensity in individual epithelial cells. (I) RT-qPCR of *Frmd5, Jak2, Stat3, Bcl2, Bcl-XL, Casp3,* and *Casp8* in vaginal tissues from 2- and 4-month mice with or without Frmd5 deletion. Experiments were independently repeated at least three times. Data are presented as mean ± SEM. *P < 0.05; **P < 0.01. (J) Schematic diagram illustrating the role of Frmd5 in regulating the Jak2-Stat3 signaling pathway and epithelial apoptosis.

We used multicolor immunofluorescence to assess the *in situ* relationship between Frmd5 and the Jak2-Stat3 signaling pathway in the vaginal epithelium. Vaginal tissues from* Cdh16-Cre+*; *Frmd5^flox/flox^* and* Cdh16-Cre-*; *Frmd5^flox/flox^* mice were examined to assess Jak2, Stat3, and phosphorylated Stat3 levels in epithelial cells, using CK14 as an epithelial cell marker (Fig. 5A). Representative cell identification is shown in Fig. S4A and fluorescence intensity quantification is shown in Fig. S4B. Epithelial cells in the* Cdh16-Cre+*;* Frmd5^flox/flox^* group exhibited significantly reduced Frmd5 levels (Fig. 5B). Jak2 expression remained unchanged (Fig. 5C). Stat3 expression was significantly increased in* Cdh16-Cre+*; *Frmd5^flox/flox^* mice (Fig. 5D), but phosphorylated Stat3 (p-Stat3) at Y705 and S727 were significantly decreased (Fig. 5E, F). Notably, p-Stat3 (Y705) and p-Stat3 (S727) levels were positively correlated with Frmd5 expression in epithelial cells from both genotypes (Fig. 5G, H). These quantitative *in situ* data demonstrate that *Frmd5* depletion suppresses Stat3 phosphorylation.

To explore the mechanism by which Frmd5 modulates apoptosis in vaginal epithelial cells via the Jak2-Stat3 signaling pathway, we analyzed the mRNA levels of Jak2-Stat3-related target genes in vaginal tissues from 2- and 4-month-old mice in both control and urinary epithelial-specific *Frmd5* knockout groups. *Frmd5* mRNA was significantly decreased in vaginal tissues of* Cdh16-Cre+*; *Frmd5^flox/flox^* mice, consistent with protein data shown in Figure 3E. *Jak2* and *Stat3* mRNA levels remained stable, but pro-apoptotic *Casp3* and *Casp8* were downregulated, whereas anti-apoptotic *Bcl2* and *Bcl-XL* were upregulated in vaginal tissues of *Cdh16-Cre+*; *Frmd5^flox/flox^* mice at both 2 and 4 months (Fig. 5I). Based on these data, we propose a schematic model illustrating how Frmd5 stabilizes the Jak2-Stat3 interaction and regulate epithelial apoptosis (Fig. 5J).

### Urinary epithelium-specific knockout of *Frmd5* leads to longitudinal vaginal septum in postnatal mice

**Figure 6:**
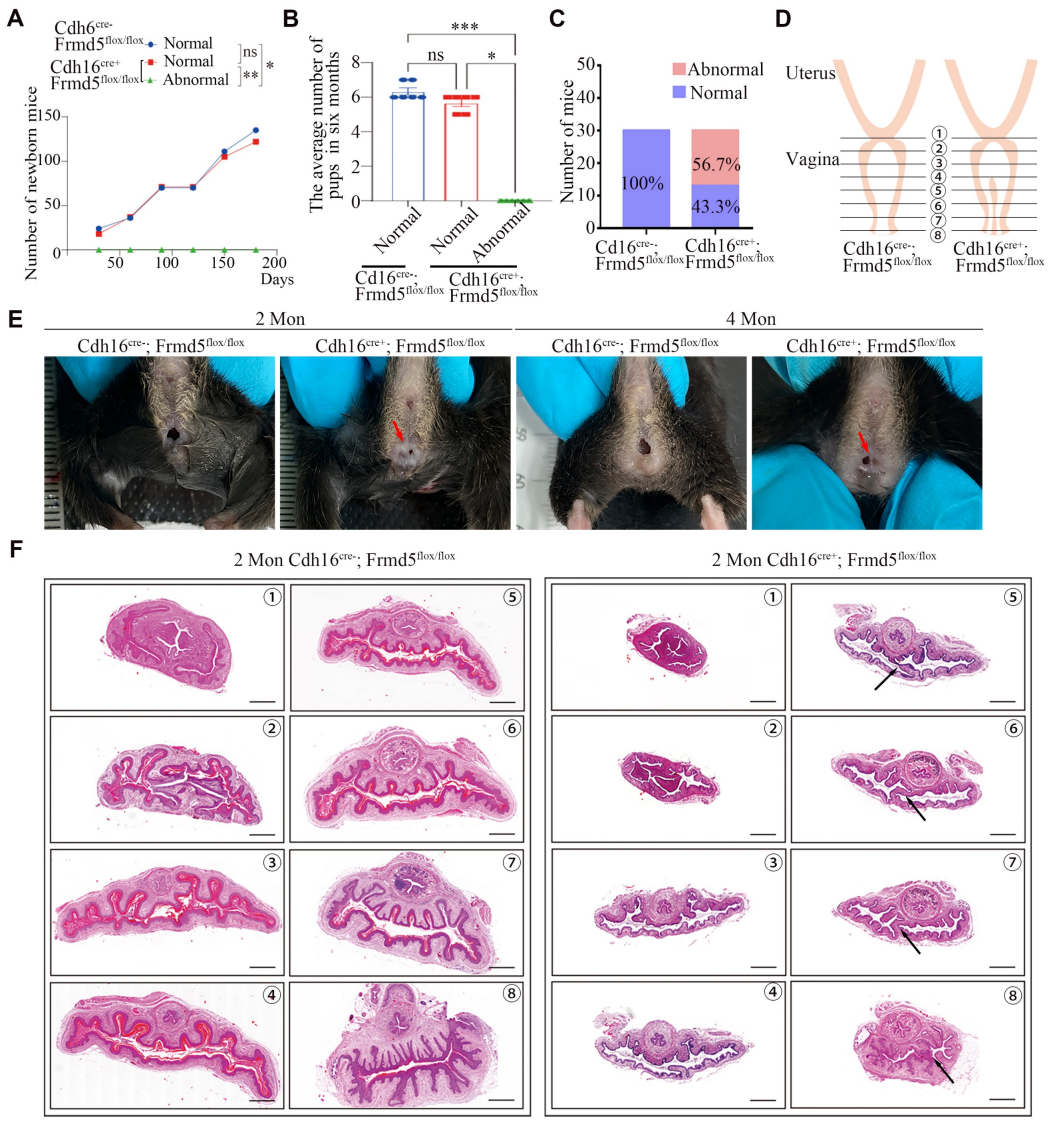
Urinary epithelium-specific knockout of *Frmd5* leads to longitudinal vaginal septum in postnatal mice. (A) Quantitative analysis of female fertility, showing the cumulative pup count over a 6-month period by genotype (*n* = 6). Statistical testing was performed by one-way analysis of variance (ANOVA). *P < 0.05, **P < 0.01. (B) Quantitative analysis of the mean litter size per pregnancy by genotype (*n* = 6). Statistical testing was performed by one-way analysis of variance (ANOVA). Data are presented as mean ± SEM. *P < 0.05, **P < 0.01, ***P < 0.001. (C) Quantitative analysis of normal versus abnormal vaginal development in* Cdh16-Cre-; Frmd5^flox/flox^* and* Cdh16-Cre+; Frmd5^flox/flox^* mice, n=30. (D) Schematic diagram of vaginal tissue morphology and consecutive sectioning positions in *Cdh16-Cre-*; *Frmd5^flox/flox^* and *Cdh16-Cre+*; *Frmd5^flox/flox^* mice. (E) Morphological observation of the vaginas in *Cdh16-Cre-*; *Frmd5^flox/flox^* and *Cdh16-Cre+*; *Frmd5^flox/flox^* mice. (F) H&E staining of vaginal tissue structure in* Cdh16-Cre-; Frmd5^flox/flox^* and* Cdh16-Cre+; Frmd5^flox/flox^* mice. Black arrows indicate the location of the longitudinal vaginal septum. Scale bar: 500 μm.

The above data show that *Frmd5* loss weakens Jak2-Stat3 activation by reducing Jak2-Stat3 interaction. We next addressed the phenotypic consequences in the urogenital tract this under Frmd5 loss. In breeding tests, we recorded the 6-month cumulative number of pups (Fig. 6A) and mean litter size (Fig. 6B) by genotype. Knockout females with a vaginal malformation were completely infertile. We found that among 30 observed *Cdh16-Cre+*; *Frmd5^flox/flox^* female mice, 43.3% (13/30) exhibited a normal vaginal opening, whereas 56.7% (17/30) displayed an incomplete opening (Fig. 6C, E).

To localize and characterize the observed vaginal malformations precisely, we prepared consecutive histological sections (positions 1-8, Fig. 6D) from *Cdh16-Cre-*; *Frmd5^flox/flox^* and *Cdh16-Cre+*; *Frmd5^flox/flox^* mice. The sections were stained with haematoxylin and eosin (H&E). Histological analysis of 2-month-old control mice revealed a tubular vaginal structure with a central, unobstructed lumen. In contrast, in *Frmd5* knockout mice, excess tissue partially occluded the lumen, forming a longitudinal septum (Fig. 6F). These data indicate that urinary epithelium-specific *Frmd5* deletion disrupts vaginal canal formation and leads to longitudinal septum formation. Because the Müllerian ducts also give rise to the uterus and oviducts, we next examined whether *Frmd5* deletion affected the morphology of these additional reproductive tissues. Histological analysis by H&E staining revealed that both the uterus and oviduct from *Cdh16-Cre+*; *Frmd5^flox/flox^* mice exhibited preserved gross structure and epithelial organization comparable to those of control mice (Figure S6).

## Discussion

In this study, we identified Frmd5, a member of the FERM domain protein family, as a novel upstream regulator of the Jak2-Stat3 signaling pathway. We demonstrated that Frmd5 stabilizes the Jak2-Stat3 complex, thereby promoting Stat3 phosphorylation and activation, which is essential for epithelial apoptosis during vaginal morphogenesis. Loss of *Frmd5* in the *Cdh16-Cre*-mediated urinary epithelium-specific knockout model led to defective canalization and the formation of a longitudinal vaginal septum, resulting in complete infertility. These findings underscore its physiological relevance of Frmd5-mediated signaling in organ development and establish a previously unrecognized regulatory layer within the Jak2-Stat3 cascade.

We investigated FRMD5, a member of the FERM domain-containing protein family known for its role as a molecular scaffold 8. These proteins mediate interactions between the extracellular matrix and intracellular signaling cascades 32, regulating embryonic development, tissue differentiation 33, platelet aggregation, and immune responses 3. Our results reveal that Frmd5 enhances Stat3 phosphorylation through stabilizing its interaction with Jak2, providing an efficient mechanism for signal amplification. It should be noted that scaffold proteins can regulate signaling through multiple non-mutually exclusive mechanisms, including enhancing spatial proximity of signaling partners, inducing conformational changes, or protecting signaling complexes from phosphatase-mediated inactivation. Although our current data cannot definitively distinguish among these possibilities, our domain-mapping results provide clues to a potential mechanism. Specifically, FRMD5 interacts with multiple STAT3 domains, including the ND+CC, DB+LK, and SH2 regions, but not with the C-terminal transactivation domain (residues 688-770) that contains the Y705 phosphorylation site. Together with our observation that Frmd5 deletion leads to reduced STAT3 Y705 phosphorylation and weakened Jak2-Stat3 interaction, these findings raise the possibility that FRMD5 may allosterically facilitate JAK2-mediated STAT3 phosphorylation by promoting a conformational arrangement that increases accessibility of the Y705 site. We emphasize that this represents a plausible mechanistic hypothesis rather than a definitive conclusion. This discovery adds a new layer of complexity to the fine-tuning of the canonical Jak-Stat signaling cascade. Interestingly, this mechanism contrasts with our previous findings on Kindlin-2, another FERM family protein, which inhibits Stat3 activation by disrupting Jak2-Stat3 binding 33. These contrasting roles highlight how different FERM proteins can exert finely tuned, even opposing, regulatory effects on the same signaling pathway. During our investigation of FRMD5-Jak2-Stat3 interactions, initial Co-IP attempts using vaginal tissue from conditional knockout mice did not produce robust signals, likely due to the relatively low proportion of epithelial cells and limited target protein abundance. We therefore used intestinal tissue from global *Frmd5* knockout mice, where the higher epithelial content enabled clear detection, and further validated our findings in *FRMD5*-knockdown HeLa cells. Together, these results establish the broader relevance of Frmd5 as a scaffold protein in Jak2-Stat3 signaling across multiple tissues.

Mechanistically, Frmd5-mediated Jak2-Stat3 signaling activation drive apoptosis by transcriptional regulating downstream targets. Loss of Frmd5 and the associated reduction in p-Stat3 (Y705) led to downregulation of pro-apoptotic genes such as* Casp3* and *Casp8*, and upregulation of anti-apoptotic genes including *Bcl2* and *Bcl-XL*. This imbalance is consistent with previous findings showing that *Bcl2* overexpression in vaginal epithelium hinders epithelial cell death and disrupts vaginal opening 30, whereas loss of *Bax* and *Bak* also causes defective canal formation 31. By linking these known apoptotic phenotypes to an upstream molecular regulator, our study fills a critical gap in understanding how epithelial apoptosis is coordinated during vaginal morphogenesis.

**Figure 7:**
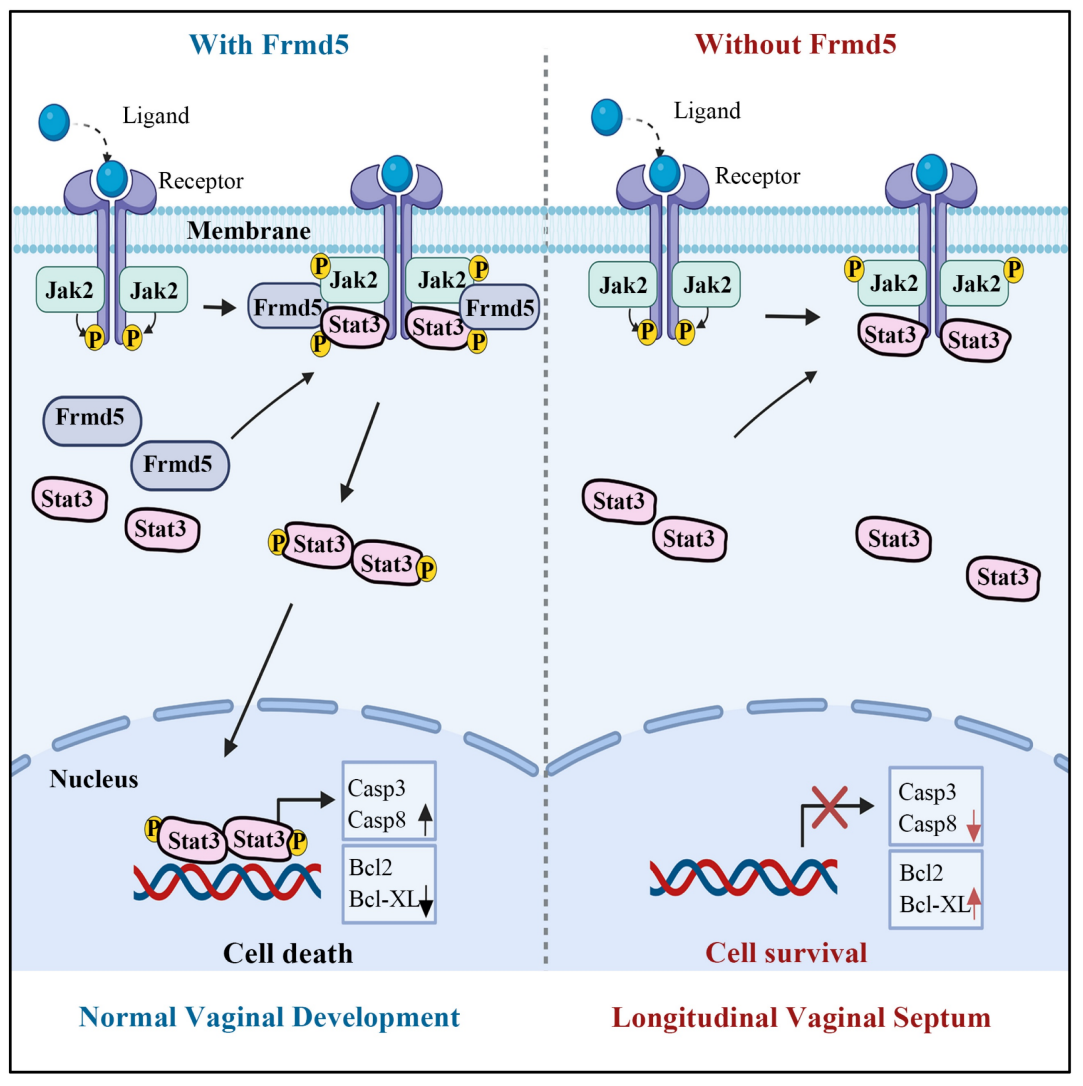
Schematic model illustrating the role of Frmd5 in mouse vaginal development.

The phenotypic manifestations observed in our knockout model provided robust physiological validation of this molecular mechanism. We selected the vagina as our model system both for its functional relevance and for methodological considerations. FRMD5, as a scaffold protein, is likely to be particularly important in tissues undergoing extensive cellular remodeling. Vaginal development, which depends heavily on apoptosis-driven remodeling, represents a classic example of such a process. Previous studies have established that the mice vagina is derived from the Müllerian ducts, with vaginal epithelial cells originating from Müllerian duct epithelium 26, 34. Mouse vaginal development initiates at embryonic day 11.5 (E11.5) with Müllerian duct differentiation 34. At birth, the vaginal canal remains closed and opens at 3-4 weeks postnatally, coinciding with puberty onset. Proper canal formation requires Müllerian duct fusion and elongation of the uterovaginal canal. Failure of duct fusion results in vaginal agenesis, incomplete elongation leads to a shortened vagina 35, and insufficient epithelial apoptosis during tissue remodeling can prevent proper vagina opening 28. Building on these developmental features, to further clarify the role of Frmd5 in vaginal development, we generated a urinary epithelium-specific Frmd5 knockout mice model using *Cdh16-Cre* and *Frmd5^flox/flox^* mice, as Cdh16 is active in Müllerian duct epithelium during embryogenesis 36, 37. Consistent with this developmental lineage, we confirmed epithelial-restricted Cdh16 expression in the vaginal epithelium at postnatal stages (Figure S5), supporting the suitability of this Cre driver for targeting vaginal epithelial cells. Moreover, the high expression of Frmd5 protein in vaginal epithelium provided an ideal system for proteomic and mechanistic analyses. Consistent with this framework, Frmd5-deficient mice exhibit longitudinal septum formation accompanied by defective epithelial remodeling. Taken together, these molecular and phenotypic findings support an integrated working model for Frmd5-mediated regulation of vaginal development (Fig. 7). In this model, Frmd5 functions as a scaffold protein that stabilizes the Jak2-Stat3 complex, thereby ensuring efficient Jak2-dependent phosphorylation of Stat3 at the Y705 site. Activated Stat3 subsequently drives a pro-apoptotic transcriptional program in vaginal epithelial cells, characterized by increased expression of Casp3 and Casp8 and suppression of anti-apoptotic factors such as Bcl2 and Bcl-XL. This coordinated apoptotic remodeling is required for timely clearance of central epithelial cells and formation of a patent vaginal lumen. In the absence of Frmd5, impaired Stat3 activation disrupts epithelial apoptosis, leading to persistence of excess epithelial cells and postnatal longitudinal septum formation.

Beyond vaginal development, our findings highlight a broader biological principle: scaffold proteins play essential roles in regulating apoptosis-driven organ morphogenesis. Infertility affects approximately one-sixth of the global population, and congenital vaginal anomalies caused by defective morphogenesis are significant contributors 21, 23. By establishing Frmd5 as a key regulator of vaginal development, our study suggests that FRMD5 dysfunction may contribute to congenital anomalies such as Mayer-Rokitansky-Küster-Hauser (MRKH) syndrome. Although this connection remains speculative, it positions FRMD5 as a promising candidate gene for further investigation in human congenital reproductive tract disorders.

Several limitations of our study should be acknowledged. While RNA-seq data indicated broader pathway perturbations, including cell cycle, growth, and adhesion, our study focused primarily on apoptosis. The full spectrum of Frmd5-regulated pathways remains to be elucidated. In addition, although we confirmed Frmd5-dependent Jak2-Stat3 stabilization in non-vaginal tissues and cultured cells, direct demonstration of this mechanism in the vaginal epithelium awaits technical advances to overcome current tissue abundance limitations. Moreover, genetic screening in patient cohorts with congenital vaginal anomalies will be necessary to determine whether FRMD5 mutations contribute to human disease. Further exploration of the nuclear dynamics and roles of the Frmd5-Stat3 complex, including whether Frmd5 and Stat3 translocate together into the nucleus to exert their function, will provide additional mechanistic insights and is an important direction for future studies.

In summary, our work establishes Frmd5 as a novel upstream scaffold of Jak2-Stat3 signaling, essential for epithelial apoptosis and vaginal canalization. These findings broaden our understanding of FERM-domain protein biology and underscore the importance of scaffold-mediated signaling in apoptosis-driven morphogenesis.

## Supplementary Material

Supplementary figures and table.

## Data Availability

The RNA-seq data reported in this paper have been deposited in the Sequence Read Archive (SRA) under accession number PRJNA1287114. The other data that support the findings of this study can be found in the manuscript, figures, and supplementary data or are available from the corresponding authors upon request.

## Abbreviations

ASD: autism spectrum disorder
BrdU: 5-bromo-2′-deoxyuridine
*Cdh16-Cre*: cadherin-16 promoter-driven Cre recombinase
Co-IP: co-immunoprecipitation
DAPI: 4′,6-diamidino-2-phenylindole
DB: DNA-binding domain
DMEM: dulbecco's modified eagle medium
EMT: epithelial-mesenchymal transition
Flag: DYKDDDDK epitope tag
FPKM: fragments per kilobase of transcript per million mapped reads
FRMD5: FERM domain-containing protein 5
GAPDH: glyceraldehyde 3-phosphate dehydrogenase
GO: gene ontology
GST: glutathione S-transferase
H&E: haematoxylin and eosin
HEK293T: human embryonic kidney 293T
HeLa: human cervical cancer cell line
HRP: horseradish peroxidase
IHC: immunohistochemistry
KEGG: kyoto encyclopedia of genes and genomes
MRKH: Mayer-Rokitansky-Küster-Hauser
PBS: phosphate-buffered saline
PCR: polymerase chain reaction
qPCR: quantitative polymerase chain reaction
RT-qPCR: quantitative reverse-transcription polymerase chain reaction
RIPA: radioimmunoprecipitation assay
RNA-seq: RNA sequencing
SD: standard deviation
SDS-PAGE: sodium dodecyl sulphate polyacrylamide gel electrophoresis
SEM: standard error of the mean
TBST: tris-buffered saline with Tween-20
TUNEL: terminal deoxynucleotidyl transferase dUTP nick-end labelling
CC: coiled-coil domain
ND: N-terminal domain
CT: C-terminal transactivation domain
SH2: Src homology 2 domain
LK: linker domain
