# Supplementary material for: Loss of Frmd5 Inhibits Jak2-Stat3 Signalling Pathway and Impairs Cell Apoptosis During Vagina Luminal Formation in Puberty Mice
Source: Int J Biol Sci. 2026 Mar 25;22(7):3843–59. doi: 10.7150/ijbs.126669 (PMC13086079; doi:10.7150/ijbs.126669)
Supplement: Supplementary file 1 — Supplementary figures and table. [file ijbsv22p3843s1.pdf]

Supplementary Materials for

Loss of Frmd5 Inhibits Jak2-Stat3 Signalling Pathway and Impairs Cell Apoptosis During Vagina Luminal Formation in Puberty Mice

Tiantian Su[1, 2, *], Zhenbin Wang[1, *], Yunjiao Wu[1], Qianchen Li[1], Li Tian[2], Xiaowei Li[2], Jing Zhang[1], Miao Yu[1], Yuqi Xing[1], Juan Du[1], Lijun Zhao[3, #], Jun Zhan[1, 3, #], Hongquan Zhang[1, 4, #]

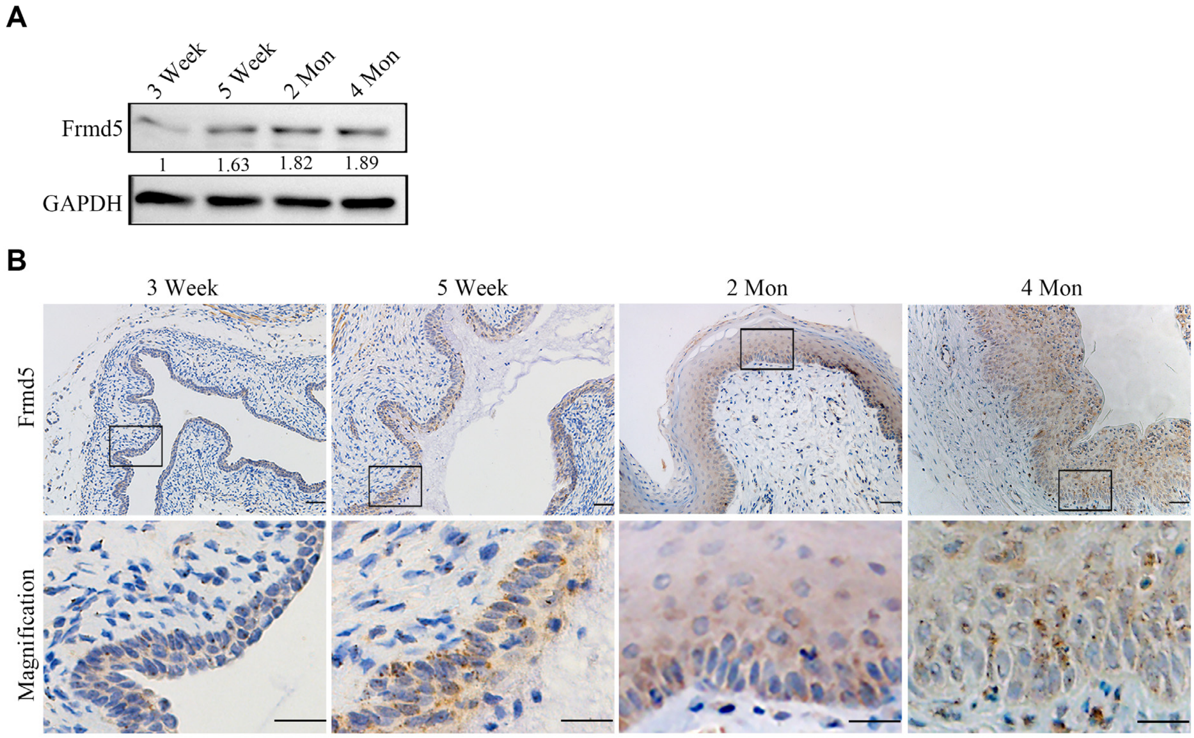

Figure S1: Distribution of Frmd5 protein in the vagina of female mice. (A) Western blot analysis of Frmd5 protein levels in the vagina at different developmental stages. (B) IHC detection of Frmd5 protein distribution in the vagina at different developmental stages. Brown staining indicates Frmd5-positive cells. Original image scale bar: 50 µm; magnified image scale bar: 20 µm.

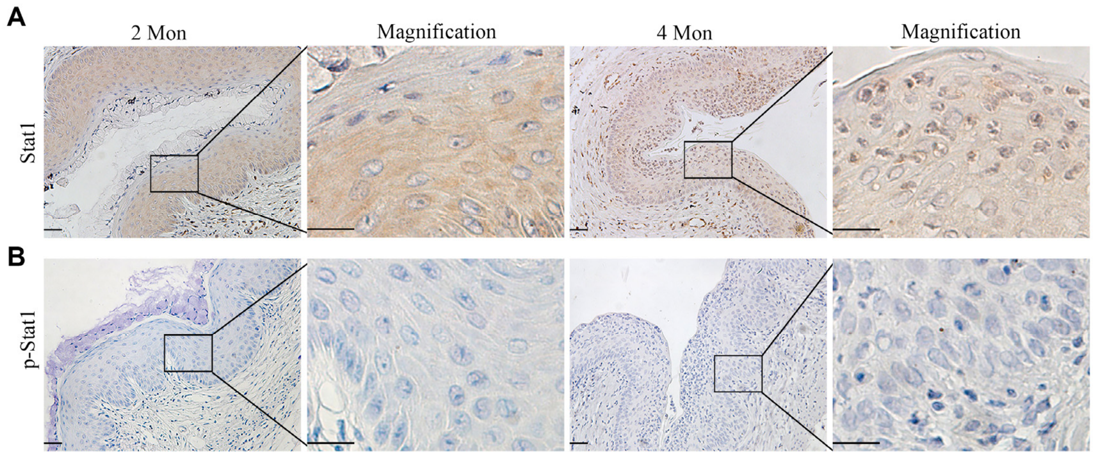

Figure S2. Distribution of Stat1 and p-Stat1 in vaginal epithelial cells of female mice. (A)

Immunohistochemical staining showing the distribution of Stat1 protein at different developmental stages, with Stat1-positive cells shown in brown. (B) Immunohistochemical staining of p-Stat1 protein at various developmental stages. Original image scale bar: 50 μm; magnified image scale bar: 20 μm.

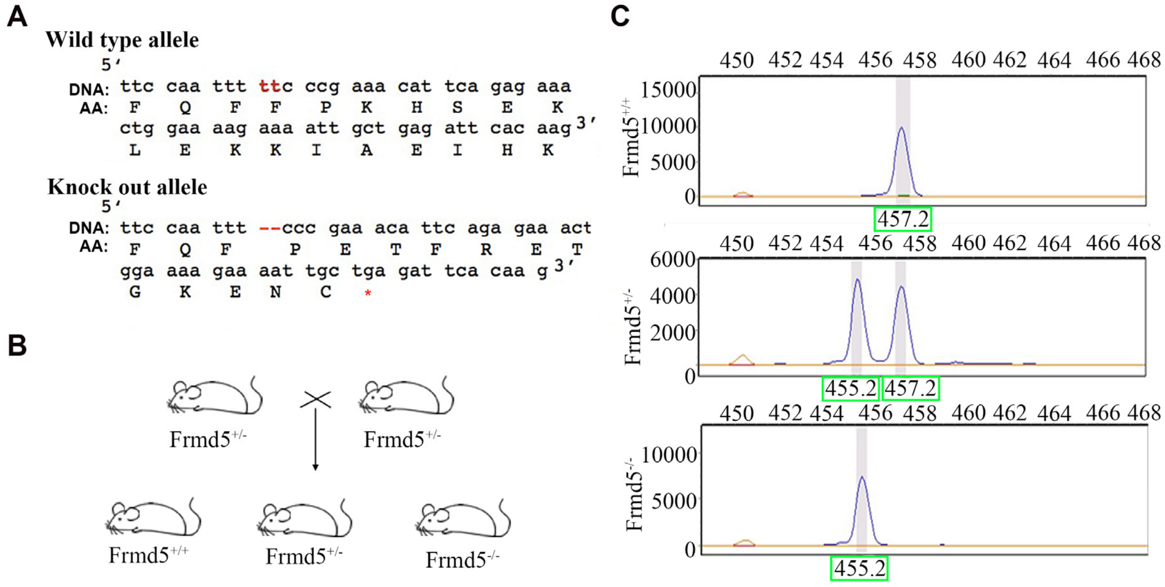

Figure S3. Generation of the global *Frmd5* knockout mice model. (A) Schematic representation of the gene-targeting strategy used to generate the global *Frmd5* knockout model. The knockout mice harbor a deletion of two nucleotides in the coding region, resulting in a frameshift mutation and the introduction of a premature stop codon shortly downstream, leading to premature translation termination. (B) Breeding strategy for generating the global *Frmd5* knockout mice. (C) Genotype confirmation of the global *Frmd5* knockout model using PCR and sequencing.

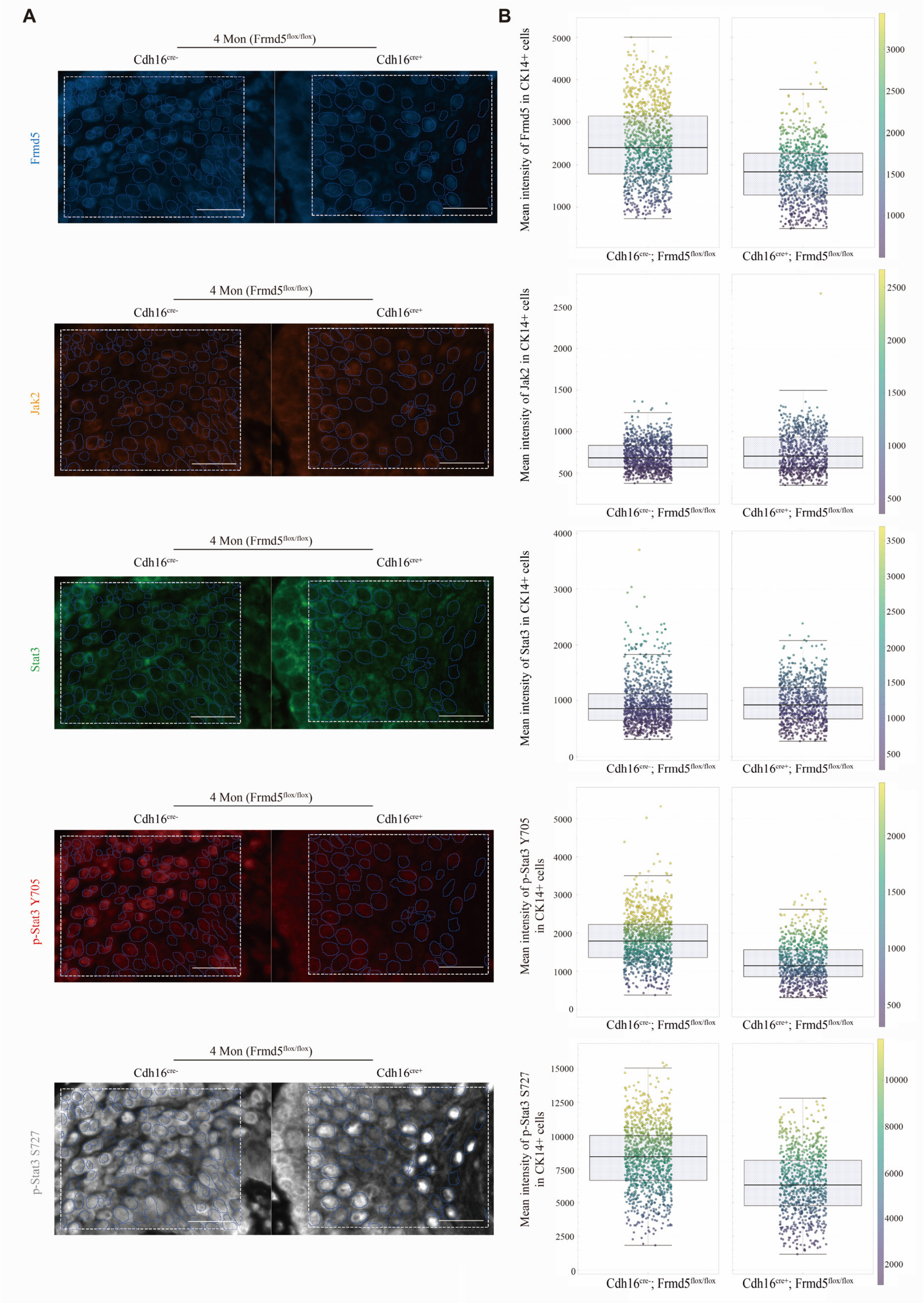

A
B
4 Mon (Frmd5flox/flox)
Cdh16cre-
Cdh16cre+
Frmd5
Jak2
Stat3
p-Stat3 Y705
p-Stat3 S727
Mean intensity of Frmd5 in CK14+ cells
Mean intensity of Jak2 in CK14+ cells
Mean intensity of Stat3 in CK14+ cells
Mean intensity of p-Stat3 Y705 in CK14+ cells
Mean intensity of p-Stat3 S727 in CK14+ cells
Cdh16cre-; Frmd5flox/flox
Cdh16cre+; Frmd5flox/flox

Figure S4. Multiplex immunofluorescence analysis of Frmd5, CK14, and Jak2-Stat3 pathway-related protein distribution. (A) Immunofluorescence images were captured using the Zeiss Axio Scan.7 system, and cell identification was performed using the Visiopharm Image Analysis System (Version: 5.0.3.1309). Dashed lines indicate the boundaries of individual cells identified by the software. (B) Fluorescence intensity analysis of all cells identified in panel A. Each point represents a single cell, with the x-axis indicating relative position of the cell and the y-axis representing fluorescence intensity.

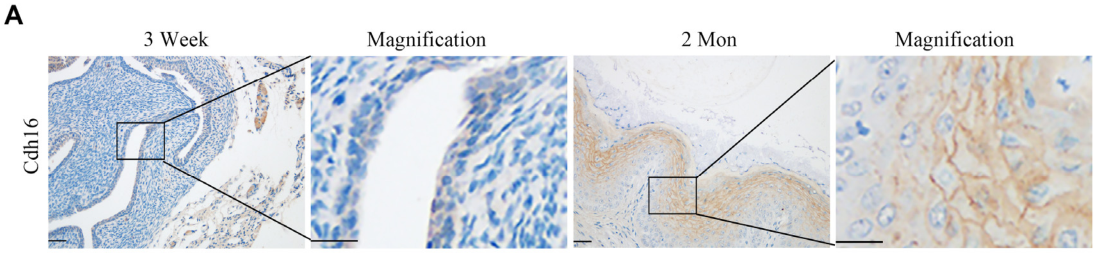

Figure S5. Distribution of Cdh16 in vaginal of female mice. (A) Immunohistochemical staining showing the distribution of Cdh16 protein at different developmental stages, with Cdh16-positive cells shown in brown. Original image scale bar: 50 µm; magnified image scale bar: 20 µm.

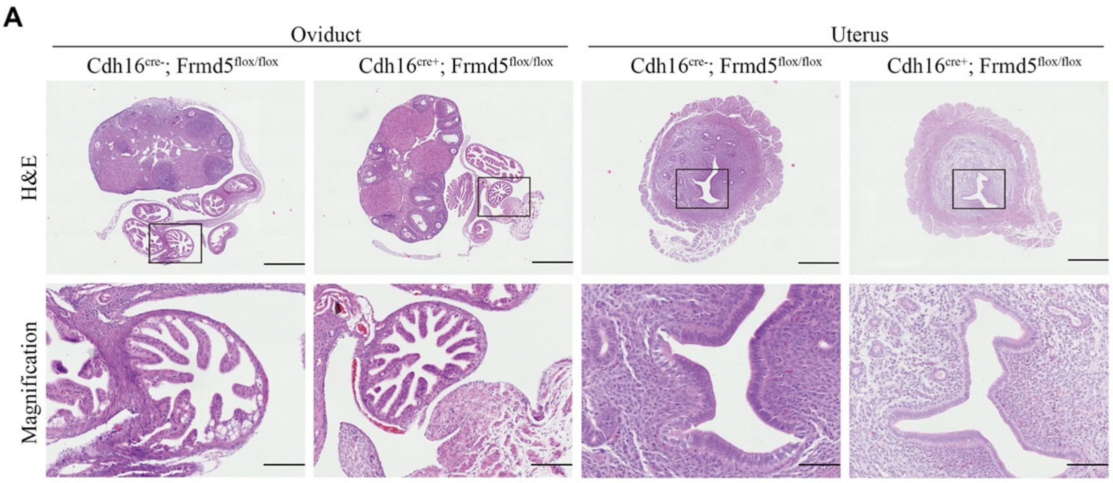

Figure S6. Histological analysis of the oviduct and uterus in *Frmd5*-deficient female mice. (A) H&E-stained sections of the oviduct and uterus from *Cdh16-Cre-; Frmd5$^{flox/flox}$* and *Cdh16-Cre+; Frmd5$^{flox/flox}$* mice. Original image scale bar: 500 µm; magnified image scale bar: 100 µm.

Gene	Forward	Reverse
Frmd5	CCTCTGGAAATGTGGAATTGAGA	CTGGACACTGTGCGGACTT
Gapdh	TGGCCTTCCGTGTTCCTAC	GAGTTGCTGTTGAAGTCGCA
Jak2	GGAATGGCCTGCCTTACAATG	TGGCTCTATCTGCTTCACAGAAT
Stat3	CACCTTGGATTGAGAGTCAAGAC	AGGAATCGGCTATATTGCTGGT
Bcl2	GCTACCGTCGTGACTTCGC	CCCCACCGAACTCAAAGAAGG
Bcl-XL	ACATCCCAGCTTCACATAACCC	CCATCCCGAAAGAGTTCATTCAC
Casp3	CTCGCTCTGGTACGGATGTG	TCCCATAAATGACCCCTTCATCA
Casp8	TGCTTGGACTACATCCCACAC	GTTGCAGTCTAGGAAGTTGACC
Xaf1	TGACACGTATTCGGGATGAAAG	GTGGGAAGATCAGCCCTCTG
Il1b	GAAATGCCACCTTTTGACAGTG	TGGATGCTCTCATCAGGACAG
Cxcl2	AGGGCGGTCAAAAAGTTTGC	CGAGGCACATCAGGTACGAT
Smox	TCCCACGGGAATCCTATCTATC	GCCACGGTTGGTAAGGTAGC
Il1rn	TAGACATGGTGCCTATTGACCT	TCGTGACTATAAGGGGCTCTTC
Ucp3	CTGCACCGCCAGATGAGTTT	ATCATGGCTTGAAATCGGACC
Ptgs1	ATGAGTCGAAGGAGTCTCTCG	GCACGGATAGTAACAACAGGGA
Msx2	CTAAAGGCGGTGACTTGTTTTCG	CGGCTTCTTGTCGGACATGAG

Table S1. Primers for RT-qPCR in mice vagina.
